# Value of Preoperative Systemic Immune-Inflammation Index and Albumin-Bilirubin Grade in Patients with Hepatocellular Carcinoma Undergoing Transarterial Embolization

**DOI:** 10.5152/tjg.2023.22296

**Published:** 2023-04-01

**Authors:** Fushuang Ha, Xue Wang, Tao Han, Kefeng Jia, Sen Wang, Dezhao Song

**Affiliations:** 1 The Third Central Clinical College of Tianjin Medical University, Tianjin, China; 2 Tianjin Key Laboratory of Extracorporeal Life Support for Critical Diseases, Tianjin, China; 3 Artificial Cell Engineering Technology Research Center, Tianjin, China; 4 Tianjin Institute of Hepatobiliary Disease, Tianjin, China; 5 Tianjin Union Medical Center, Tianjin Medical University, Tianjin, China

**Keywords:** Albumin-bilirubin grade, hepatocellular carcinoma, overall survival, prognosis, systemic immune-inflammation index, transarterial chemoembolization

## Abstract

**Background:**

**:** The systemic immune-inflammation index reflects the systematic inflammatory status, and the albumin-bilirubin grade reflects the liver function. In patients with hepatocellular carcinoma receiving transarterial chemoembolization, their combined clinical utility has not been fully explored. Herein, we purposed to determine the prognostic worthiness of systemic immune-inflammation index–albumin-bilirubin scores in patients receiving transarterial chemoembolization for unresectable hepatocellular carcinoma.

**Methods:**

Patients who were treated with transarterial chemoembolization after being diagnosed with hepatocellular carcinoma between 2008 and 2016 were recruited for this research work. Systemic immune-inflammation index and albumin-bilirubin scores were determined prior to treatment. The clinico-pathological factors related to overall survival were determined via univariate along with multivariate analyses.

**Results:**

A total of 295 patients were retrospectively studied. Patients with systemic immune-inflammation index–albumin-bilirubin score of 2 had the worst outcomes, exhibiting a median overall survival of 11 months (95% CI, 8.44-13.56 months) in contrast with subjects in the systemic immune-inflammation index–albumin-bilirubin 1 group (median OS, 26 months; 95% CI, 21.25-30.75 months) and the systemic immune-inflammation index–albumin-bilirubin 0 class (median OS, 31 months; 95% CI, 12.76-49.24 months). The 1-, 3-, and 5-year rates of survival were 45.3%, 1.3%, and 0% for patients in the systemic immune-inflammation index–albumin-bilirubin 2 category; 76.4%, 35.0%, and 14.6% for those in the systemic immune-inflammation index–albumin-bilirubin 1 category; and 85.6%, 46.7%, and 35.0% for those in the systemic immune-inflammation index–albumin-bilirubin 0 category, respectively (*P < .*001).

**Conclusions:**

The systemic immune-inflammation index–albumin-bilirubin score could be a simple indicator to estimate the prognosis in individuals with hepatocellular carcinoma being treated with transarterial chemoembolization. Patients in the systemic immune-inflammation index–albumin-bilirubin 2 category were more likely to be related to a shorter overall survival.

**Main Points**

- Patients treated with transarterial chemoembolization (TACE) have diverse clinical outcomes owing to remarkably heterogeneous characteristics of tumor burden coupled with liver functional reserve. Thus, a potential prognostic indicator is needed to identify patients with potentially poor prognosis after TACE.
- The purpose of this research work was to determine if combining systemic immune-inflammation index with albumin-bilirubin I grade improves prognostic accuracy.
- This study exhibited that for individuals with hepatocellular carcinoma (HCC) who underwent TACE, the systemic immune-inflammation index–albumin-bilirubin (SII-ALBI) score at baseline could be a simple indicator to estimate the prognosis. Subjects with HCC in the SII-ALBI 2 category were more likely to have a shorter overall survival.

## Introduction

Hepatocellular carcinoma (HCC) is the sixth most frequent cancer globally and the fourth primary cause of cancer-linked deaths.^1^ In most cases, HCC can be prevented by resection because of the tumor location and size or liver dysfunction. Transarterial chemoembolization (TACE) has been suggested as the first line of therapy for Barcelona Clinic Liver Cancer (BCLC) B stage HCC, some BCLC A stage HCC cases, and may also benefit BCLC-C stage HCC individuals with moderate liver function.^2^ However, patients treated with TACE have diverse clinical outcomes owing to remarkably heterogeneous characteristics of tumor burden coupled with liver functional reserve. Thus, a potential prognostic indicator is needed to identify patients with potentially poor prognosis after TACE. Several investigations described 2 markers of HCC prognosis, the systemic immune-inflammation index (SII) along with the albumin-bilirubin (ALBI) grade.

Systemic immune-inflammation index may indicate how well the host immunological and inflammatory responses are balanced.^1^ The SII is computed as follows: SII = peripheral platelet counts × peripheral neutrophil counts/peripheral lymphocyte counts.^1^ An elevated SII is closely related to the prognosis of many solid tumors, such as HCC.^1,2^ Some investigations have documented a relationship of elevated SII with dismal prognosis in individuals with HCC being treated with TACE.^3,4^

The Child-Turcotte-Pugh (CTP) score, which was developed in 1964 and later updated to evaluate prognosis following surgery for variceal bleeding in individuals with cirrhosis, is the most extensively used model to estimate liver functional state.^5,6^ Many HCC staging systems, including the BCLC staging approach, use CTP categorization for assessment of the severity of the liver disease.^7^ However, the CTP score harbors some limitations consisting of subjective factors (encephalopathy along with ascites) and interrelated factors (serum albumin and ascites) and has not been statistically proven.^8^ Recently, ALBI grade was proposed as an optional measurement model of liver function based only on albumin along with bilirubin.^9^ ALBI = (albumin × −0.085) + (log_10_ bilirubin × 0.66), in which the units of bilirubin are micromole per liter and that of albumin is gram per liter.^9^ There are 3 grades in ALBI: ALBI > −1.39 is grade 3, ALBI >−2.60 to ≤−1.39 is grade 2, and ALBI ≤−2.60 is grade 1.^9^ Albumin-bilirubin grade has been verified in several investigations with diverse stages of the disease and numerous medications, including TACE, and has proved to be a valuable tool for objectively assessing liver function along with treatment outcome in individuals with HCC.^10-13^

To date, no research work has attempted to assess the predictive worthiness of combining inflammatory along with liver function markers in individuals with HCC receiving TACE. Thus, the purpose of this research work was to determine if combining SII with ALBI grade improves prognostic accuracy.

## Materials and Methods

This research work was granted approval by the Ethics Committee of the Third Central Clinical College of Tianjin Medical University. In this premise, all procedures involving human participants were as per the Declaration of Helsinki, 1964. All subjects granted written informed consent prior to TACE.

### Study Design and Patient Selection

A review of the electronic medical records of subjects with newly diagnosed HCC who had been treated with TACE between March 2008 and December 2016 at our hospital was done. Diagnosis of HCC was done via contrast-enhanced dynamic computed tomography or magnetic resonance imaging exhibiting early hyper-enhancement in arterial phase, as well as delayed washout in the venous phase.^14-16^ All individuals with HCC enrolled in this research work were classified as BCLC stage A, B, and C. Comprehensive demographic information consisting of HCC etiology, the burden of tumor, status of liver function, and laboratory data along with performance status was gathered. Subject enrollment criteria consisted of (1) monotherapy with TACE as the initial treatment; (2) Eastern Cooperative Oncology Group performance status 0-1; and (3) CTP A or B liver function. Subject exclusion criteria consisted of the following: (1) had diffused HCC; (2) had incomplete baseline data; (3) survival was <3 months; (4) underwent hepatectomy or liver transplantation; (5) underwent other local treatments; (6) underwent systemic therapy; (7) severe underlying cardiac and renal diseases; (8) known active or chronic infection at blood sampling time; and (9) had other types of cancer. Hepatocellular carcinoma linked to hepatitis B virus (HBV) was characterized by individuals who tested positive for the HBV surface antigen (HBsAg). Individuals who tested positive for anti-hepatitis C virus (HCV) antibodies were regarded to have HCC caused by HCV. Alcoholism was described as daily alcohol consumption of at least 40 g for men and 20 g for women, over a period of more than 5 years. All the patients with HBsAg or HCV-RNA positive received antiviral treatment. All the patients were informed about systematic treatments if they were eligible for treatment. Patients who accepted systemic therapy were excluded from this study. Patient baseline data were recorded 1–7 days before TACE. At our center, portal vein thrombosis and major vascular invasion in subjects with good hepatic function are not regarded as absolute contraindications to TACE. Patient clinical data were reviewed and analyzed according to confidentiality requirements.

### Defining the Combined Inflammation and Liver Function Grade

The optimal cut-off value of SII was determined via receiver operating characteristic (ROC) curve assessments, on the basis of ROC curve most prominent point for “sensitivity” along with “1−specificity,” respectively. Next, using the Youden index (maximum [sensitivity+specificity − 1]), the appropriate cut-off value was computed.^17^ Thereafter, a new grading system for inflammation and liver function, the SII–ALBI score, was developed by combining the SII with the ALBI grades. The SII–ALBI score was calculated as follows: patients in whom SII was elevated on the basis of the ROC curve assessment and ALBI was grade 2 or 3 were designated a score of 2; subjects exhibiting an elevation in one or neither of these parameters were designated a score of 1 or 0, respectively (Table 1).

### TACE Procedure and Follow-up

Two senior interventional radiologists with comparable expertise in the management of HCC executed uniform TACE. Under local anesthesia, the Seldinger approach was adopted in accessing the right femoral artery. With a selective or supra-selective injection, a mixture of fluorodeoxyuridin, cisplatin and/or pirarubicin in ≤20 mL lipiodol was injected, followed by embolization with polyvinyl alcohol particles or gelatin sponge. The chemotherapeutic agent dose was computed on the basis of the body surface area. If new nodules, enlarged lesions, or elevated low lipiodol uptake were observed, TACE was repeated after an interval of 1.5–3.0 months. Treatment was terminated if a subject could not tolerate the therapy owing to a decline in clinical status or if a complete response was achieved. A follow-up of all patients post-TACE was performed until death or the cut-off date (July 31, 2021).

### Statistical Analysis

Data analyses were implemented in Statistical Package for the Social Sciences version 25.0 (IBM Corp.; Armonk, NY, USA). Continuous variables are given as means ± standard deviations or median with range. Categorical data are given as frequencies and were analyzed via the Pearson’s χ^2^ test or Fisher’s exact test. To identify the optimal SII cut-off values, ROC curve evaluations were performed. Overall survival was estimated from the start of TACE treatment to death or the end of the final follow-up period. Overall survival was computed via the Kaplan-Meier approach, and the survival curve equivalences were established through log-rank statistics. We conducted univariate along with multivariate Cox regression analyses to establish the possible prognostic markers. Statistical significance was signified by *P* < .05.

## Results

### Optimal Cut-off Value for the SII

**Figure 1.**
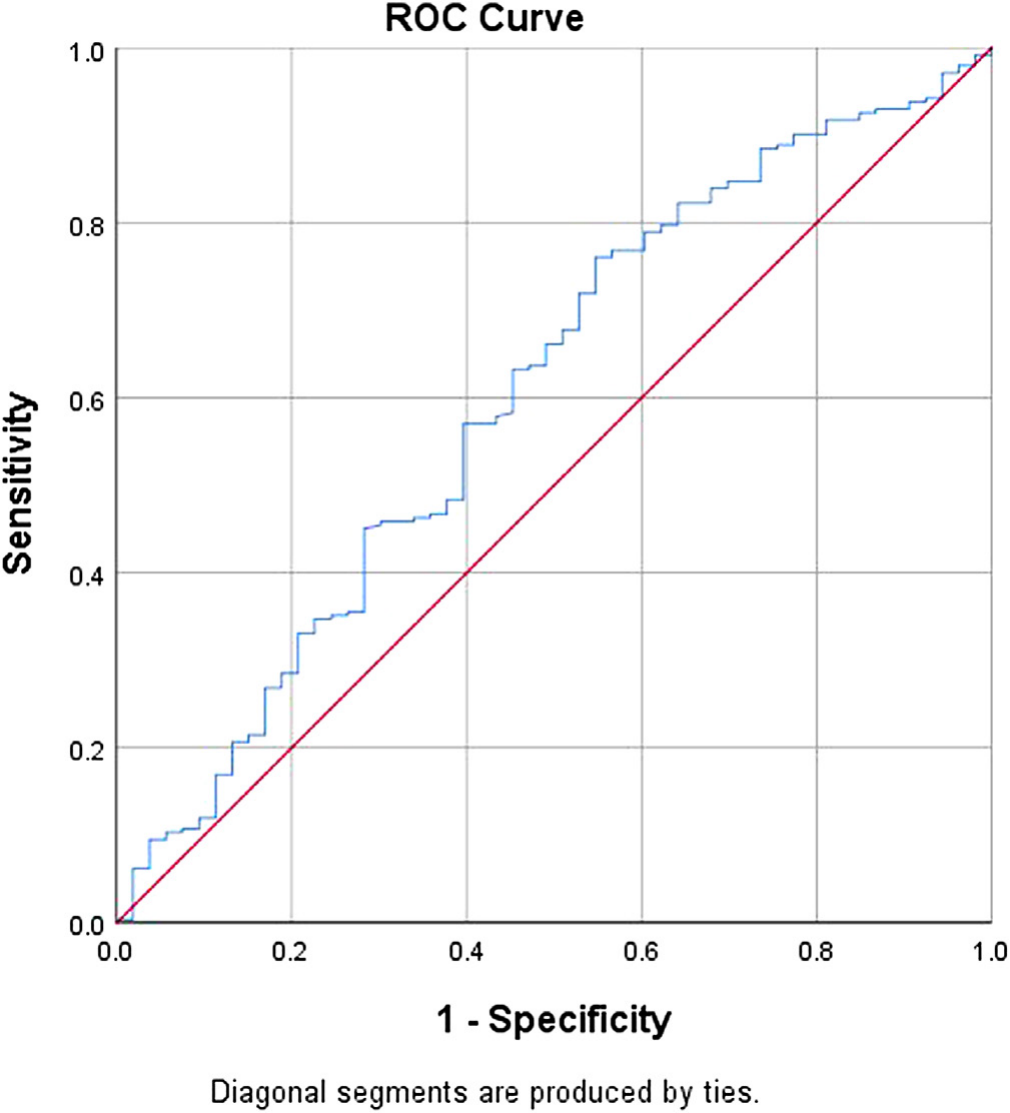
Receiver operating characteristic curves to assess the best cutoff value of systemic immune-inflammation index. AUROC: 0.602; 95% CI: 0.515-0.689; *P = .*019, with a sensitivity of 0.756 and specificity of 0.453.

The optimal cut-off value for SII was 152.80, harboring a sensitivity of 0.756 along with specificity of 0.453 (AUROC curve: 0.602; 95% CI: 0.515-0.689) (Figure 1).

### Baseline Characteristics

Overall, 295 subjects with HCC were treated with TACE monotherapy as initial treatment and were enrolled in this research premise between March 2008 and December 2016. There were 251 males (85.1%) and 44 females (14.9%). On the basis of the BCLC stage, 71 patients were stage A, 119 were stage B, and 105 were stage C. On the basis of the ALBI grade, 91 patients were categorized as level 1 (30.8%), 192 as level 2 (65.1%), and 12 as level 3 (4.1%). A total of 283 (95.9%) patients had cirrhosis.

### Associations between SII-ALBI Grade and Clinicopathological Features of Individuals with HCC

Among the 295 subjects, 15 (5.1%) harbored an SII–ALBI score of 0, while 143 (48.5%) and 137 (46.4%) harbored an SII–ALBI score of 1 and 2, respectively. The SII–ALBI 2 class had a remarkably higher alpha-fetoprotein (AFP) level, a bigger largest size of the tumor, and a higher BCLC C stage versus the other groups (*P < .*05). Table 2 summarizes the clinico-pathological characteristics along with demographic features of subjects on the basis of the SII–ALBI score.

### Survival Analysis

**Figure 2.**
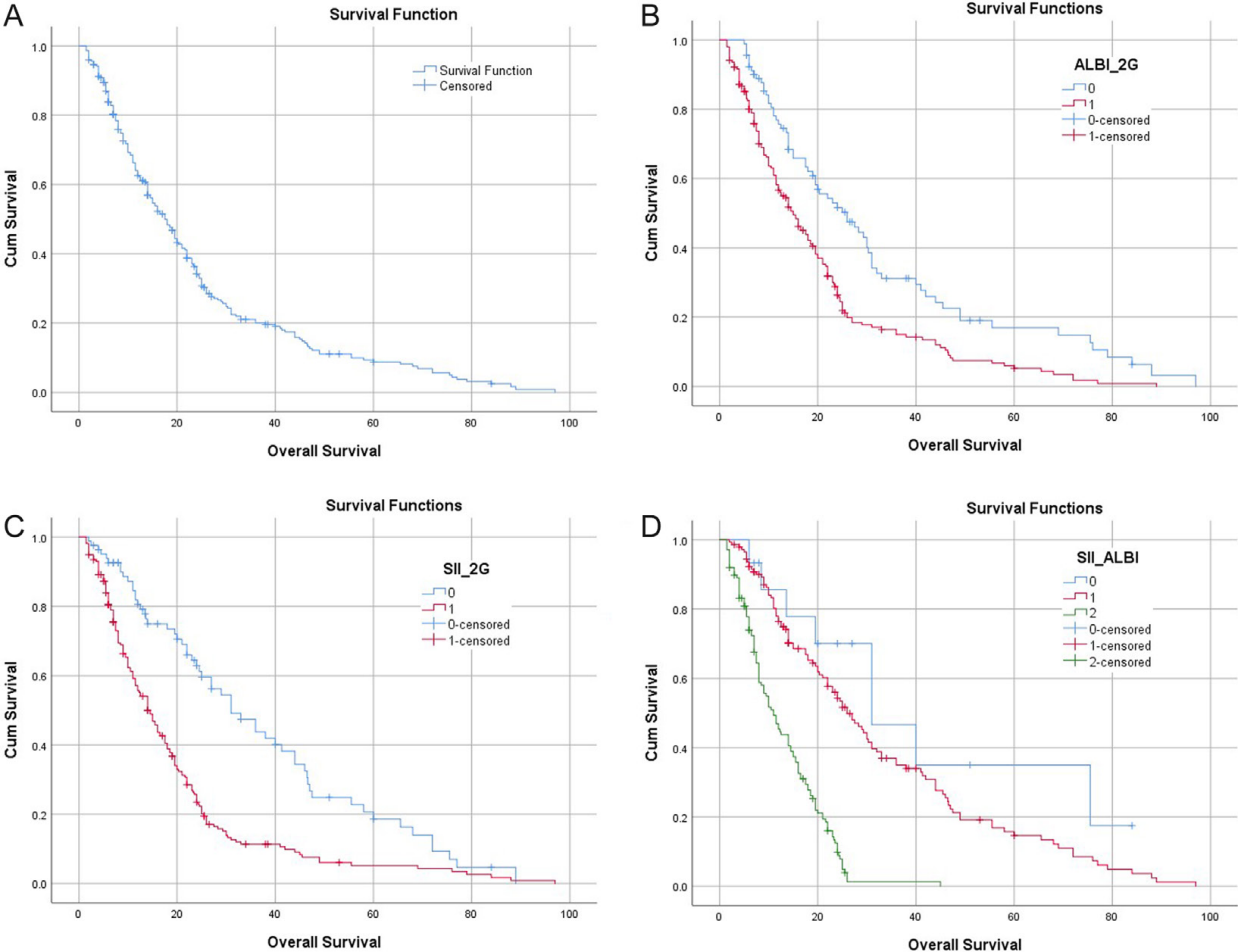
Overall survival curves in HCC subjects being treated with transarterial chemoembolization for hepatocellular carcinoma via the Kaplan-Meier approach. (A) Overall survival; (B) SII; (C) ALBI grade; and (D) SII-ALBI. HCC, hepatocellular carcinoma; SII-ALBI, systemic immune-inflammation index along with albumin-bilirubin scores.

During the follow-up period, a total of 242 participants (82.0%) died. The median length of survival was 17.5 months (95% CI, 15.0-20.0 months). The rates of OS at 1-, 3-, and 5 years were 62.6%, 20.0%, and 8.8%, respectively (Figure 2A). The 295 individuals with HCC were stratified into 2 groups on the basis of their SII score, that is, SII ≤152.80 (n* = *82) and SII >152.80 (n* = *213). On the basis of the Kaplan-Meier approach, the median OS for subjects with a SII152.80 was 31 months (95% CI, 22.18-39.82 months), in contrast with 14 months (CI, 11.76-16.24 months) for those with an SII >152.80. Besides, the 1-, 3-, and 5-year OS rates of subjects with an SII of 152.8 were remarkably greater in contrast with those with an SII of 152.80 (80.5%, 43.8%, and 18.6, respectively, vs. 55.6%, 11.4%, and 5.2%, *P < .*001) (Figure 2B). The data suggested that a high SII level is related to a poor prognosis in individuals with unresectable HCC.

Similarly, we stratified patients on the basis of their AIBL grade: ALBI grade 1 (n *= *91) or ALBI grades 2 and 3 (n* = *204). The median OS for individuals with ALBI grade 1 was remarkably longer versus those with ALBI grade 2 or 3 (26 months, 95% CI, 18.07-33.93 months vs. 15 months, 95% CI, 11.80-18.20 months, respectively, *P < .*001). Additionally, the 1-, 3-, and 5-year overall rates of survival for subjects with ALBI grade 1 were remarkably higher in contrast with those for subjects with ALBI grades 2 and 3 (75.7%, 31.1%, and 16.9%, respectively, vs. 56.6%, 15.0%, and 5.3%, *P < .*001) (Figure 2C). A high ALBI grade reflected a dismal prognosis in individuals with HCC receiving TACE.

Lastly, subjects with SII–ALBI score of 2 had the worst outcomes, harboring a median OS of 11 months (95% CI, 8.44-13.56 months) in contrast with those in the SII–ALBI 1 class (median OS, 26 months; 95% CI, 21.25-30.75 months) and the SII–ALBI 0 class (median OS, 31 months; 95% CI, 12.76-49.24 months). The 1-, 3-, and 5-year rates of survival were 45.3%, 1.3%, and 0% for patients in the SII-AIBI 2 class; 76.4%, 35.0%, and 14.6% for those in the SII–ALBI 1 class; and 85.6%, 46.7%, and 35.0% for those in the SII–ALBI 0 class, respectively (*P < .*001) (Figure 2D). Thus, our data indicated that pre-operative SII–ALBI scores of 2 were related to the worst survival.

## Risk Factors for Outcome after Transarterial Chemoembolization

Table 3 summarizes the findings of univariate along with the multivariate analyses of the determinants of post-operative OS. Pre-operative alanine aminotransferase (ALT), aspartate aminotransferase (AST), total bilirubin, AFP, tumor number, BCLC stage, SII, and ALBI were all linked with favorable post-operative outcomes (*P < .*05). Multivariate assessment indicated that the following variables were remarkable independent predictors of OS: AST [*P = .*041; HR 1.333 (1.011-1.757)], AFP [*P < .*001; HR 1.800 (1.342-2.413)], BCLC stage [*P < .*001; HR 2.570 (1.936-3.413)], SII [*P < .*001; HR 3.472 (2.415-4.992)], and ALBI [*P < .*001; HR 2.592 (1.808-3.716)] (Table 3).

## Discussion

Transarterial chemoembolization constitutes the standard treatment for BCLC-B stage HCC patients and some patients in BCLC-A stage with contraindications to resection and thermal ablation.^18^ However, given its impacts on enhancing survival and decreasing financial burden, numerous BCLC-C stage HCC subjects with compensatory hepatic function also undergo TACE. The prognosis of patients after TACE varies widely given the high heterogeneity among patients. Hence, we believe that simple and useful markers to precisely estimate the prognosis of patients being treated with TACE are needed. Herein, we found that combining inflammation and liver function-based SII–ALBI is an independent prognostic indicator for estimating the survival of patients undergoing TACE monotherapy as the initial treatment. Moreover, SII–ALBI 2 category was established to be related to a relatively dismal prognosis following TACE.

This study has demonstrated that a high SII (>152.80) independently predicted shorter survival in patients undergoing TACE. Congruent with our data, SII values were also documented to be a predictive risk factor for patients with HCC following treatments other than TACE, for instance, hepatectomy, sorafenib therapy, or liver transplantation.^1,19,20^

systemic immune-inflammation index constitutes a systemic inflammatory index that employs neutrophil, lymphocyte, along with platelet counts to precisely indicate the degree of systemic inflammation. Besides, inflammation is a remarkable factor in the tumor microenvironment. Neutrophilia could repress immune cells, for instance, lymphocytes, activated T cells, as well as natural killer cells, so impairing the immune system.^21,22^ Lymphocytes are pivotal elements of the adaptive immune system because they are responsible for immunosurveillance along with immunoediting functions. Lymphocyte invasion was demonstrated to be a sign of an efficient anti-tumor cellular immune response.^23^ The entrance of cancer cells into the circulation initiates platelet recognition, which is increased by cell surface receptors, immune cells, cellular products, and extracellular factors. In other cases, these cross-talks dampen the immune system recognition and removal of cancer cells or increase endothelial arrest, as well as trapping in the microvasculature and survival.^24^

The ALBI grade is a new assessment approach for hepatic function, which is considered to be better relative to the CTP score for assessing hepatic function along with treatment outcomes in individuals with HCC via numerous studies.^10-13^ The CTP score was inferior with regards to stability, because it included subjective factors (encephalopathy and ascites) and had inter-related factors (serum albumin and ascites), and it was not statistically proven.^8^ In contrast, ALBI grade is a more objective and simple method to assess liver function, which may be better to evaluate individuals with HCC.^25^ Hepatocellular carcinoma subjects with relatively poorly preserved hepatic function are associated with more treatment-related toxicity, shorter survival, slower recovery, and increased complications.^26^

We hypothesized that combining SII with ALBI would enhance the accuracy of predictive evaluation for patients with HCC who underwent TACE, because the combination could assess both the inflammation status and the hepatic function of these patients.

Herein, 295 subjects with newly diagnosed HCC were included. The TACE monotherapy was performed as the initial treatment for all the patients. All the patients with HBsAg or HCV-RNA positivity received antiviral treatment, because studies suggest that antivirals are beneficial to the management of hepatitis-B-related HCC and sustained virological response after oral direct-acting antivirals therapy may result in improved liver dysfunction and facilitate additional HCC-directed therapy.^27,28^ The multivariate along with univariate analyses results exhibited that baseline SII and ALBI were independent influencing factors for the OS of patients with HCC who underwent TACE.

For the baseline data, SII–ALBI score had a better discriminative potential when compared with either SII or ALBI alone. Systemic immune-inflammation index–albumin-bilirubin 2 category was associated with relatively higher AFP levels, bigger largest tumor size, more BCLC C stage, and worse OS. The results of this study can help to establish patients who had a potentially poor prognosis after TACE in order to modify the treatment plan and give timely systematic treatment.

To the best of our knowledge, this is the first research work to evaluate the prognostic value of SII–ALBI combination in patients with HCC who underwent TACE. However, this research premise had some limitations. This was a retrospective study from a single center, which may lead to potential bias because of the limited sample size. So, a multi-center and prospective cohort study with a large sample size is necessary to verify the prognostic value of SII–ALBI score in HCC after TACE and the potential underlying mechanism.

## Conclusion

This study exhibited that for individuals with HCC who underwent TACE, the SII-ALBI score at baseline could be a simple indicator to estimate the prognosis. Subjects with HCC in the SII-ALBI 2 category were more likely to have a shorter OS. Hence, SII–ALBI score should be considered when formulating or adjusting the treatment plan in individuals with HCC being treated with TACE.

## Figures and Tables

**Table 1.** The Combination of SII and ALBI as Prognostic Indices

Variable	Score
SII	
≤152.80	0
>152.80	1
ALBI	
Level 1	0
Levels 2 and 3	1
SII-ALBI	
SII = 0 and ALBI = 0	0
SII = 1 or ALBI =1	1
SII = 1 and ALBI = 1	2

SII-ALBI, systemic immune-inflammation index along with albumin-bilirubin scores.

**Table 2.** Comparison of the Clinical Characteristics of Patients with Different SII-ALBI Scores

Variable	SII-ALBI (n* = *15)	SII-ALBI 1 (n = 143)	SII-ALBI 2 (n = 137)	*P*
Age, years	57.2 ± 8.63	58.80 ± 9.32	59.20 ± 9.08	.712
Gender (male/female)	11/4	119/24	121/16	.206
Etiology (Hepatitis B/Hepatitis C/ Alcoholic hepatitis/Others)	10/1/0/4	81/18/16/28	79/6/15/37	.154
ALT, IU/L	39.60 ± 20.83	44.63 ± 33.29	47.69 ± 27.57	.503
AST, IU/L	30.73 ± 14.84	38.95 ± 34.49	48.23 ± 37.79	.037^*^
TBIL, mmol/L	18.69 ± 6.05	21.86 ± 12.80	26.43 ± 12.66	.003^*^
ALB, g/L	42.78 ± 2.39	38.56 ± 5.42	34.03 ± 4.84	<.001^*^
Platelet count (×10^9^L^−1^)	69 ± 39.42	110.99 ± 79.33	115.10 ± 63.5	.059
Neutrophil count (×10^9^L^−1^)	2.10 ± 0.84	2.79 ± 1.38	3.19 ± 1.21	.001^*^
Lymphocyte count (×10^9^L^−1^)	1.37 ± 0.89	1.20 ± 0.57	0.93 ± 0.49	<.001^*^
Creatinine (mmol/L)	65.4 ± 14.87	64.87 ± 14.11	65.45 ± 16.64	.950
Child-Pugh score	5.27 ± 0.59	6.05 ± 1.37	6.78 ± 1.42	<.001^*^
MELD score	4.72 ± 3.09	4.73 ± 4.24	5.99 ± 3.85	.007^*^
Largest tumor size (cm)	57.73 ± 33.07	53.03 ± 33.23	65.52 ± 53.79	.020^†^
AFP (≤400/>400)	12/3	111/32	92/45	.039^†^
Tumor number (single/multiple)	10/5	74/69	42/95	<.001^*^
Vascular invasion (−/+)	10/5	104/39	89/48	.368
Esophageal varices (−/+)	3/12	51/92	35/102	.124
Liver cirrhosis (−/+)	0/15	10/133	2/135	.046^*^
BCLC stage (A and B/C)	10/5	100/43	80/57	.045^†^

^†^ *P < .*05 for SII-ALBI 2 versus the other 2 groups.

^∗^ *P < .*05 for each group compared with other groups.

SII-ALBI, systemic immune-inflammation index along with albumin-bilirubin scores; BCLC, Barcelona Clinic Liver Cancer; TBIL, total bilirubin; MELD, The Model for End-Stage Liver Disease.

**Table 3.** Prognostic Factors Associated with OS

Variables	Univariate		Multivariate	
Age, years (≤60, >60)	0.836(0.643-1.086)	0.180		
Gender (male/female)	0.686(0.467-1.008)	0.055		
ALB, g/L (≤35, >35)	0.827(0.638-1.072)	0.152		
TBIL, mmol/L (≤28, >28)	1.393(1.052-1.845)	0.021		
ALT, IU/L (≤40, >40)	1.356(1.053-1.747)	0.018		
AST, IU/L (≤35, >35)	1.450(1.125-1.869)	0.004	1.345(1.019-1.774)	0.036
AFP, ng/mL (≤400, >400)	1.881(1.414-2.501)	<0.001	1.813(1.352-2.432)	<0.001
MELD score (≤10, >10)	1.414(0.956-2.093)	0.083		
Largest tumor size, cm (≤3, >3)	1.219(0.905-1.642)	0.192		
Tumor number (single/multiple)	1.629(1.250-2.124)	<0.001		
Liver cirrhosis (+/−)	1.491(0.763-3.915)	0.243		
Esophageal varices (+/−)	1.154(0.878-1.516)	0.304		
BCLC stage (A and B vs. C)	2.689(2.052-3.524)	<0.001	2.608(1.965-3.463)	<0.001
SII (≤152.80, >152.80)	2.211(1.636-2.988)	<0.001	3.372(2.354-4.830)	<0.001
ALBI (levels 1, 2, & 3)	1.820(1.368-2.422)	<0.001	2.521(1.766-3.598)	<0.001

SII-ALBI, systemic immune-inflammation index along with albumin-bilirubin scores; BCLC, Barcelona Clinic Liver Cancer.

## References

[1] Hu B Yang XR Xu Y et al. Systemic immune-inflammation index predicts prognosis of patients after curative resection for hepatocellular carcinoma. Clin Cancer Res. 2014;20(23):6212 6222. (10.1158/1078-0432.CCR-14-0442) 25271081

[2] Wang H Lin C Fan W et al. Dynamic changes in the neutrophil-to-lymphocyte ratio predict the prognosis of patients with hepatocellular carcinoma undergoing transarterial chemoembolization. Cancer Manag Res. 2020;12:3433 3444. (10.2147/CMAR.S245396) 32523374 PMC7234956

[3] Yang Z Zhang J Lu Y et al. Aspartate aminotransferase-lymphocyte ratio index and systemic immune-inflammation index predict overall survival in HBV-related hepatocellular carcinoma patients after transcatheter arterial chemoembolization. Oncotarget. 2015;6(40):43090 43098. (10.18632/oncotarget.5719) 26506519 PMC4767493

[4] Zhao LY Yang DD Ma XK et al. The prognostic value of aspartate aminotransferase to lymphocyte ratio and systemic immune-inflammation index for Overall Survival of Hepatocellular Carcinoma Patients Treated with palliative treatments. J Cancer. 2019;10(10):2299 2311. (10.7150/jca.30663) 31258733 PMC6584423

[5] Child CG Turcotte JG . Surgery and portal hypertension. Major Probl Clin Surg. 1964;1:1 85. 4950264

[6] Pugh RN Murray-Lyon IM Dawson JL Pietroni MC Williams R . Transection of the oesophagus for bleeding oesophageal varices. Br J Surg. 1973;60(8):646 649. (10.1002/bjs.1800600817) 4541913

[7] Llovet JM Brú C Bruix J . Prognosis of hepatocellular carcinoma: the BCLC staging classification. Semin Liver Dis. 1999;19(3):329 338. (10.1055/s-2007-1007122) 10518312

[8] Hiraoka A Kumada T Michitaka K Kudo M . Newly proposed ALBI grade and ALBI-T score as tools for assessment of hepatic function and prognosis in hepatocellular carcinoma patients. Liver Cancer. 2019;8(5):312 325. (10.1159/000494844) 31768342 PMC6873026

[9] Johnson PJ Berhane S Kagebayashi C et al. Assessment of liver function in patients with hepatocellular carcinoma: a new evidence-based approach-the ALBI grade. J Clin Oncol. 2015;33(6):550 558. (10.1200/JCO.2014.57.9151) 25512453 PMC4322258

[10] Hansmann J Evers MJ Bui JT et al. Albumin-bilirubin and platelet-albumin-bilirubin grades accurately predict overall survival in high-risk patients undergoing conventional transarterial chemoembolization for hepatocellular carcinoma. J Vasc Interv Radiol. 2017;28(9):1224 1231.e2. (10.1016/j.jvir.2017.05.020) 28688815

[11] Kao WY Su CW Chiou YY et al. Hepatocellular carcinoma: nomograms based on the albumin-bilirubin grade to assess the outcomes of radiofrequency ablation. Radiology. 2017;285(2):670 680. (10.1148/radiol.2017162382) 28562211

[12] Zhao S Wang M Yang Z et al. Comparison between Child-Pugh score and albumin-bilirubin grade in the prognosis of patients with HCC after liver resection using time-dependent ROC. Ann Transl Med. 2020;8(8):539. (10.21037/atm.2020.02.85) PMC7214905 32411762

[13] Antkowiak M Gabr A Das A et al. Prognostic role of albumin, bilirubin, and ALBI scores: analysis of 1000 patients with hepatocellular carcinoma undergoing radioembolization. Cancers. 2019;11(6). (10.3390/cancers11060879) PMC6627853 31238514

[14] Wang Y Peng C Cheng Z et al. The prognostic significance of preoperative neutrophil-lymphocyte ratio in patients with hepatocellular carcinoma receiving hepatectomy: a systematic review and meta-analysis. Int J Surg. 2018;55:73 80. (10.1016/j.ijsu.2018.05.022) 29787804

[15] Marrero JA Kulik LM Sirlin CB et al. Diagnosis, staging, and management of hepatocellular carcinoma: 2018 practice guidance by the American Association for the Study of liver diseases. Hepatology. 2018;68(2):723 750. (10.1002/hep.29913) 29624699

[16] Ronot M Purcell Y Vilgrain V . Hepatocellular carcinoma: current imaging modalities for diagnosis and prognosis. Dig Dis Sci. 2019;64(4):934 950. (10.1007/s10620-019-05547-0) 30825108

[17] Youden WJ Index for rating diagnostic tests. Cancer. 1950;3(1):32 35. (10.1002/1097-0142(1950)3:1<32::aid-cncr2820030106>3.0.co;2-3) 15405679

[18] Vogel A Martinelli E clinicalguidelines@esmo.org, ESMO Guidelines Committee. Updated treatment recommendations for hepatocellular carcinoma (HCC) from the ESMO Clinical Practice Guidelines. Ann Oncol. 2021;32(6):801 805. (10.1016/j.annonc.2021.02.014) 33716105

[19] Fu H Zheng J Cai J et al. Systemic Immune-Inflammation Index (SII) is useful to predict survival outcomes in patients after liver transplantation for hepatocellular carcinoma within Hangzhou criteria. Cell Physiol Biochem. 2018;47(1):293 301. (10.1159/000489807) 29768257

[20] Hong YM Yoon KT Cho M . Systemic immune-inflammation index predicts prognosis of sequential therapy with sorafenib and regorafenib in hepatocellular carcinoma. BMC Cancer. 2021;21(1):569. (10.1186/s12885-021-08124-9) PMC8130266 34006248

[21] el-Hag A Clark RA . Immunosuppression by activated human neutrophils. Dependence on the myeloperoxidase system. J Immunol. 1987;139(7):2406 2413. 2821114

[22] Petrie HT Klassen LW Kay HD . Inhibition of human cytotoxic T lymphocyte activity in vitro by autologous peripheral blood granulocytes. J Immunol. 1985;134(1):230 234. 3871101

[23] Liu C Jia BS Zou BW et al. Neutrophil-to-lymphocyte and aspartate-to-alanine aminotransferase ratios predict hepatocellular carcinoma prognosis after transarterial embolization. Medicine. 2017;96(45):e8512. (10.1097/MD.0000000000008512) PMC5690744 29137051

[24] Menter DG Tucker SC Kopetz S Sood AK Crissman JD Honn KV . Platelets and cancer: a casual or causal relationship: revisited. Cancer Metastasis Rev. 2014;33(1):231 269. (10.1007/s10555-014-9498-0) 24696047 PMC4186918

[25] Peng Y Qi X Guo X . Child-Pugh versus score for the assessment of prognosis in Liver Cirrhosis: a systematic review and meta-analysis of observational studies. Medicine. 2016;95(8):e2877. (10.1097/MD.0000000000002877) PMC4779019 26937922

[26] Zou H Yang X Li QL Zhou QX Xiong L Wen Y . A comparative study of albumin-bilirubin score with Child-Pugh score, model for end-stage liver disease score and indocyanine green R15 in predicting posthepatectomy liver failure for hepatocellular carcinoma patients. Dig Dis. 2018;36(3):236 243. (10.1159/000486590) 29495004

[27] Fouad M Abdel-Rahman O . Combination of transarterial chemoembolisation (TACE) plus antivirals for the management of hepatitis B-related hepatocellular carcinoma: a systematic review of the literature. Arab J Gastroenterol. 2015;16(2):40 45. (10.1016/j.ajg.2015.03.003) 25910573

[28] Singal AG Lim JK Kanwal F . AGA clinical practice update on interaction between oral direct-acting antivirals for chronic hepatitis C infection and hepatocellular carcinoma: expert review. Gastroenterology. 2019;156(8):2149 2157. (10.1053/j.gastro.2019.02.046) 30878469 PMC6529246

